# Global extracellular vesicle proteomic signature defines U87-MG glioma cell hypoxic status with potential implications for non-invasive diagnostics

**DOI:** 10.1007/s11060-019-03262-4

**Published:** 2019-08-14

**Authors:** Vineesh Indira Chandran, Charlotte Welinder, Kelin Gonçalves de Oliveira, Myriam Cerezo-Magaña, Ann-Sofie Månsson, Maria C. Johansson, Gyorgy Marko-Varga, Mattias Belting

**Affiliations:** 1 Department of Clinical Sciences, Lund, Division of Oncology and Pathology, Lund University, Lund, Sweden; 2 Department of Biomedical Engineering, Clinical Protein Science & Imaging, Biomedical Center, Lund University, Lund, Sweden; 3 Department of Hematology, Oncology and Radiophysics, Skåne University Hospital, Lund, Sweden; 4 Department of Immunology, Genetics and Pathology, Uppsala University, Uppsala, Sweden

**Keywords:** Glioblastoma, Hypoxia, Extracellular vesicles, Mass spectrometry, Label free quantification

## Abstract

**Purpose:**

Glioblastoma multiforme (GBM) is the most common and lethal of primary malignant brain tumors. Hypoxia constitutes a major determining factor for the poor prognosis of high-grade glioma patients, and is known to contribute to the development of treatment resistance. Therefore, new strategies to comprehensively profile and monitor the hypoxic status of gliomas are of high clinical relevance. Here, we have explored how the proteome of secreted extracellular vesicles (EVs) at the global level may reflect hypoxic glioma cells.

**Methods:**

We have employed shotgun proteomics and label free quantification to profile EVs isolated from human high-grade glioma U87-MG cells cultured at normoxia or hypoxia. Parallel reaction monitoring was used to quantify the identified, hypoxia-associated EV proteins. To determine the potential biological significance of hypoxia-associated proteins, the cumulative *Z* score of identified EV proteins was compared with GBM subtypes from HGCC and TCGA databases.

**Results:**

In total, 2928 proteins were identified in EVs, out of which 1654 proteins overlapped with the ExoCarta EV-specific database. We found 1034 proteins in EVs that were unique to the hypoxic status of U87-MG cells. We subsequently identified an EV protein signature, “HYP_SIGNATURE_”, encompassing nine proteins that strongly represented the hypoxic situation and exhibited close proximity to the mesenchymal GBM subtype.

**Conclusions:**

We propose, for the first time, an EV protein signature that could comprehensively reflect the hypoxic status of high-grade glioma cells. The presented data provide proof-of-concept for targeted proteomic profiling of glioma derived EVs, which should motivate future studies exploring its utility in non-invasive diagnosis and monitoring of brain tumor patients.

**Electronic supplementary material:**

The online version of this article (10.1007/s11060-019-03262-4) contains supplementary material, which is available to authorized users.

## Introduction

Glioblastoma multiforme (GBM) is the most common and malignant type of primary brain tumor in adults with a median survival of approximately 15 months [1–3]. GBM is identified from less malignant, low grade gliomas, by extensive regions of hypoxia [4] that directly correlate with the aggressive behaviour [5]. Hypoxia results from the high proliferative and metabolic activity of malignant cells [6] and is associated with pseudopalisading necrosis as well as vascular hyperproliferation [7]. Tumor hypoxia modulates stromal cell interactions in the microenvironment that further support the survival and dissemination of malignant cells [4, 8–11]. Numerous studies have previously shown that tumor progression is driven by hypoxic signaling [12], and the expression of hypoxia-related markers correlate with poor patient outcome in several tumor types, including GBM [13]. However, the development of strategies for non-invasive monitoring of brain tumor hypoxic signalling remains a challenge of high clinical relevance, especially with regard to the relative inaccessibility and spatiotemporal heterogeneity of GBM tumors.

Extracellular vesicles (EVs) are excessively secreted by tumor cells into the circulation, and are emerging as a promising candidate for liquid biopsy-based approaches in cancer [14–16]. Exosomes and microvesicles are lipid-bilayer EVs [17] that have come to be recognized in intercellular communication, promoting the development and progression of various disease conditions [18]. Numerous studies have shown that exosome-like EVs may mediate hypoxia-dependent intercellular signaling in GBM [19]. Moreover, pilot studies based on an antibody array targeted at angiogenesis-related proteins, suggested that the EV proteome may reflect the tumor oxygenation status in GBM [20]. To further develop EV-based strategies for non-invasive tumor diagnosis and monitoring of hypoxia, it is essential to comprehensively identify proteins that are efficiently sorted to EVs and that reflect the hypoxic status of the cell or tissue of origin.

In this study, we employed label free quantification (nontargeted method) and parallel reaction monitoring (targeted method) to globally characterize the proteome of EVs derived from U87-MG high-grade glioma cells with the aim to understand how EV profiling can be exploited to noninvasively define the hypoxic status of glioma tumors.

## Results

### Global proteome identification in EVs derived from high-grade glioma cells

**Fig. 1:**
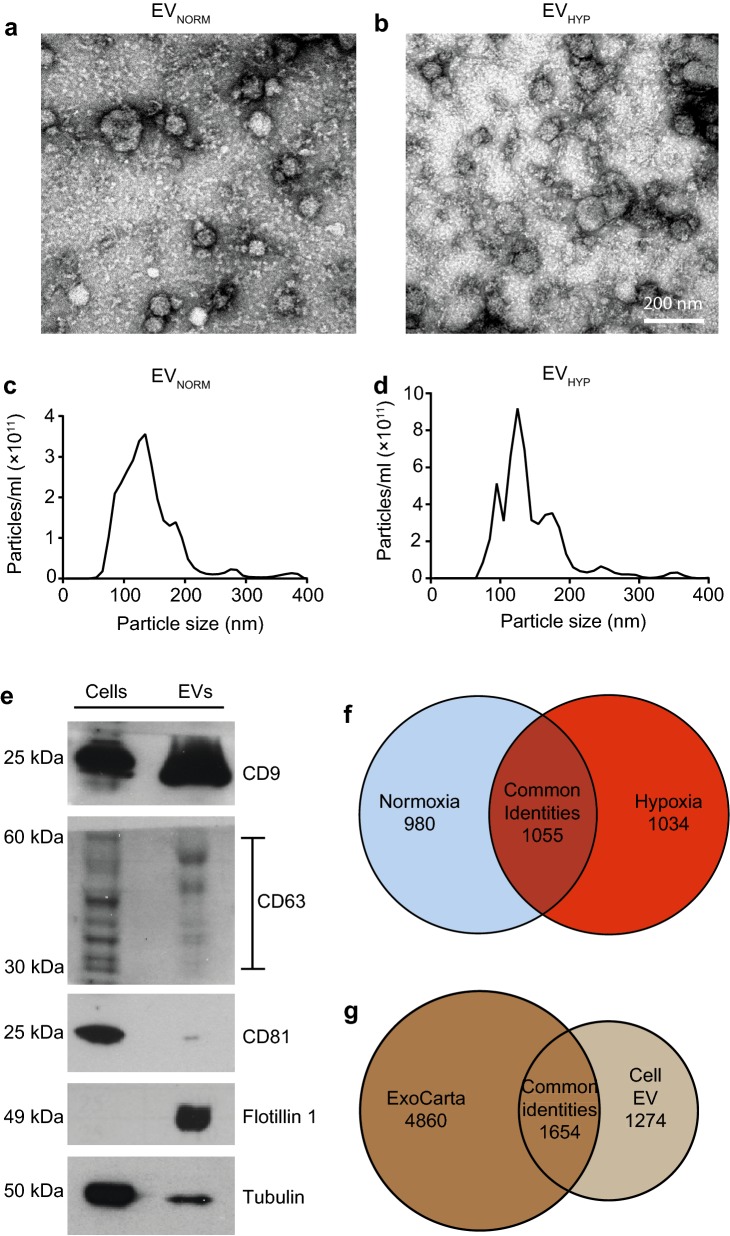
Characterization of EVs isolated from normoxic and hypoxic glioma cells. Electron microscopy shows comparable shape and size distribution of EVs isolated from normoxic (**a**) and hypoxic (**b**) U87-MG cells. Scale bar, 200 nm. Nano tracking analysis showing variations in particle concentration in normoxia (**c**) and hypoxia (**d**) of U87-MG cell-derived EVs. **e** U87-MG cells and EVs were probed for CD9, CD63, CD81, Flotillin 1, and tubulin by immunoblotting. **f** Venn diagram illustrating protein groups identified in normoxic (*n* = 9) and hypoxic (*n* = 12) cell EVs using LC–MS/MS procedures, as indicated in the Supplementary Methods section. **g** Venn diagram showing comparison of protein groups identified in U87-MG cell EVs by LC–MS/MS with ExoCarta, a public EV proteomics database

EVs from U87-MG, i.e. the most well-characterized human glioma cell-line [21, 22], grown under normoxic (EV_NORM_) or hypoxic (EV_HYP_) conditions were isolated by standard sequential ultracentrifugation [20]. The size distribution and morphology of EVs was analyzed by transmission electron microscopy (TEM), where EV_NORM_ and EV_HYP_ predominantly were found in the size range of 50–150 nm in diameter with no apparent difference in their morphology (Fig. 1a, b). Nanoparticle tracking analysis (NTA) showed similar size distribution, where both EV_NORM_ and EV_HYP_ were found in the size range of 80–150 nm (Fig. 1c, d), which is consistent with the typical size distribution profile of exosomes [23]. We found significantly increased secretion of EVs by U87-MG cells when cultured under hypoxia as compared to normoxia (Fig. 1d), which is in accordance with previous findings [24, 25]. Currently, in addition to the mechanism of biogenesis and size [26], EVs are generally referred to as exosomes also based on the expression of CD9, CD63, and CD81 proteins [27], which were all found to be present in U87-MG derived EVs, together with a strong enrichment of the membrane raft marker Flotillin 1 (Fig. 1e).

We then employed shotgun proteomics by data-dependent acquisition to comprehensively determine the proteome of EV_NORM_ and EV_HYP_ derived from U87-MG cells. We identified a total of 2089 EV_HYP_ and 2035 EV_NORM_ proteins (Fig. 1f; Supplementary Tables 1, 2). There were 1034 protein groups unique to EV_HYP_ (Fig. 1f; Supplementary Table 3) and 1055 protein groups common to both EV_NORM_ and EV_HYP_ (Fig. 1f; Supplementary Table 4). We next created a multiconsensus list combining EV_NORM_ and EV_HYP_ protein identities (Supplementary Table 5) and then compared the multiconsensus protein group to the ExoCarta EV public database [28]. The multiconsensus EV identities (2928 proteins) showed extensive overlapping of 1654 common identities with the ExoCarta database and also identified 1274 unique identities (Fig. 1g), which support the sensitivity of detection of the EV proteome with the current approach.

### Processing of the EV proteome by label free quantification (LFQ)

**Fig. 2:**
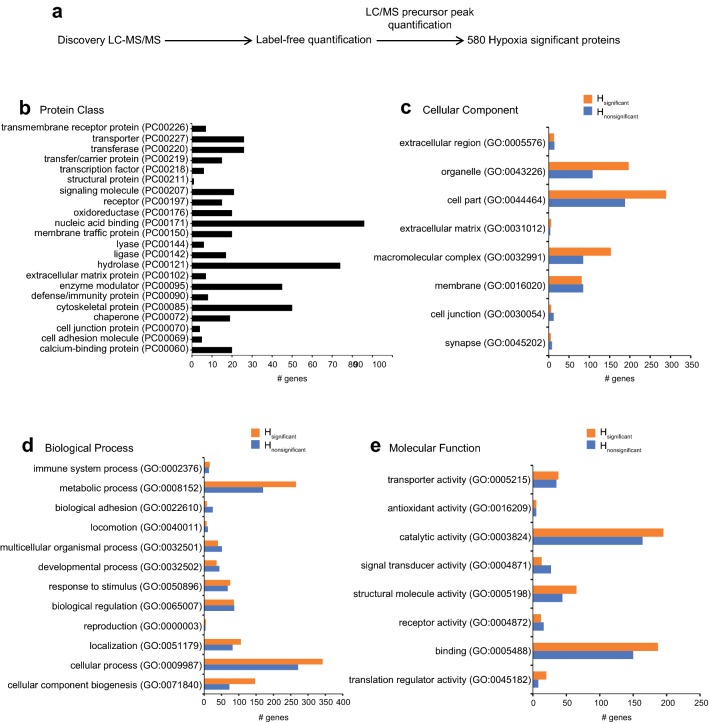
Non-targeted label free quantification of EV protein groups identified by LC–MS/MS. Schematic diagram showing label free quantification of EV proteins identified by Proteome Discoverer (**a**). Functional classification analyses assigned as protein class (**b**), cellular component (**c**), biological process (**d**), and molecular function (**e**) of H_significant_ (orange bars) and H_nonsignificant_ (blue bars) proteins

Discovery MS analysis resulted in the identification of thousands of proteins, and it is not feasible to analyze the abundance signature of each individual protein by targeted MS/MS. Therefore, to filter the proteins identified in EV_NORM_ and EV_HYP_ based on their significance in hypoxia, we subjected the discovery MS-identified proteins to nontargeted LFQ in Proteome Discoverer (PD) version 2.2 (Fig. 2a). We could then obtain the abundance value of each protein in EV_HYP_ and EV_NORM_ in terms of the LC/MS precursor peak quantification of the unique peptides for a particular protein. Subsequently, a ratio of the abundance values of each protein in EV_HYP_ over EV_NORM_ was calculated, which identified a total of 580 hypoxia significant (H_significant_) proteins (Log2 fold change, cut-off > 0.01), and other proteins that were above Log2 fold change cut-off > 0.01, were taken as hypoxia downregulated (H_nonsignificant_) proteins (Supplementary Table 6).

H_significant_ proteins were found to be distributed mostly in nucleic acid binding, hydrolase, enzyme modulators, and cytoskeletal protein subclasses, as determined by Gene Ontology system of classification using PANTHER version 14.0 [29] (Fig. 2b). Then we analysed the differences in functional classification pertaining to biological processes, molecular functions, and cellular localization of the H_significant_ (orange bars) and H_nonsignificant_ (blue bars) proteins (Fig. 2). A substantially higher number of H_significant_ proteins were localized in organelles (GO:0,043,226) and macromolecular complexes (GO:0,032,991) compared to H_nonsignificant_ proteins (Fig. 2c). More H_significant_ proteins were associated with cellular (GO:0,009,987), metabolic (GO:0,008,152), and cellular component biogenesis processes (GO:0,071,840) and catalytic activity (GO:0,003,824) compared to H_nonsignificant_ proteins (Fig. 2d, e), consistent with characteristics of the hypoxic tumor state [30, 31].

### Validation of H_significant_ profile by parallel reaction monitoring (PRM)

**Fig. 3:**
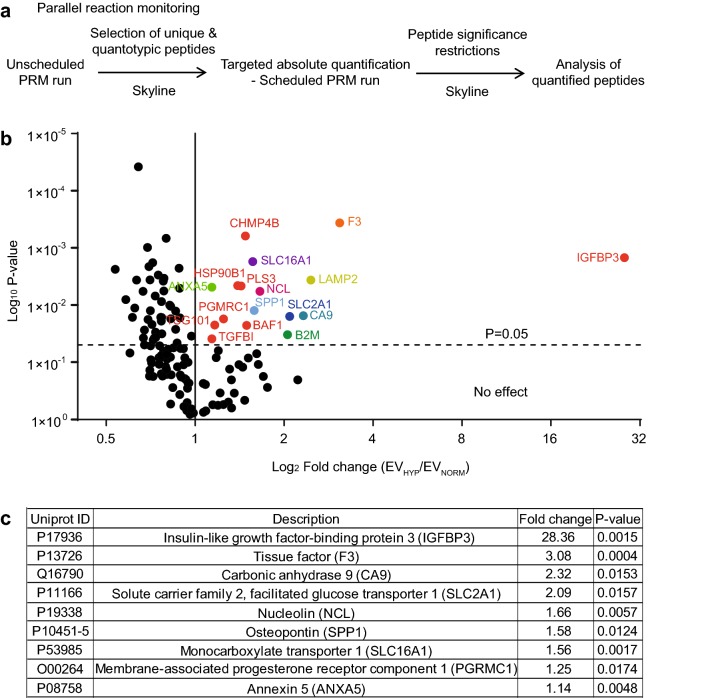
Relative quantification of selected EV protein groups/peptides by LC-PRM-MS/MS. **a** Schematic diagram showing quantification of proteome extracted from normoxic or hypoxic U87-MG cell-derived EVs by PRM. **b** Volcano plot showing differentially expressed proteins in EVs isolated from normoxic and hypoxic U87-MG cells. Each protein is represented as a dot and is mapped according to its fold change (EV_HYP_ compared to EV_NORM_) on the abscissa axis (x) and *t* test *P* value on the ordinate axis (y). Colored dots indicate significant proteins and black dots indicate non-significant proteins. **c** List of proteins found enriched (fold change) in EVs isolated from hypoxic as compared with normoxic U87-MG cells, as quantified by PRM

To validate the H_significant_ proteins identified above by LFQ, we next performed PRM (Fig. 3a). A set of selection criteria specific for targeted PRM analysis as described in Rauniyar was applied [32], including peptide length, uniqueness, miscleavage, modification, precursor charge, chromatographic peak, and signal intensity to further filter identified protein groups and select appropriate quantotypic peptides for proteins of interest using Skyline version 3.1. In addition, we added a few protein groups based on their relevance in glioma. Consequently, we selected a total of 135 protein groups with 5 unique quantotypic peptides per protein group for quantification by targeted PRM. Firstly, we performed an unscheduled PRM run on EV_NORM_ and EV_HYP_ samples to analyze the ionization of selected peptides and optimize their retention time and transition charge state. The chromatogram output was analyzed in Skyline and the 2 to 3 most quantotypic flyable peptides and appropriate transition states per protein were selected for the scheduled PRM run for all 135 protein groups (Supplementary Table 7).

The peak normalized areas (PAN) of individual peptides of all proteins analyzed were extracted from the Skyline, and the average of replicate PAN values of each individual peptide of all proteins in EV_NORM_ or EV_HYP_ samples were calculated. In all cases, the selected peptides of 135 candidate proteins had quantifiable distribution of area under curve for the identified peptide transitions (Supplementary Table 8). On analysing the fold change, we found 17 proteins significantly differentially expressed in EV_HYP_ as compared to EV_NORM_ (Fig. 3b; Supplementary Table 8). We further applied peptide significance and normalized peak area restrictions on the hypoxia response of the H_significant_ EV proteins (*N* = 17) and filtered it down to a signature of 9 proteins that included Insulin-like Growth Factor-Binding Protein 3 (IGFBP3), Tissue Factor (F3), Carbonic Anhydrase 9 (CA9), Solute Carrier Family 2 Facilitated Glucose Transporter Member 1 (SLC2A1), Nucleolin (NCL), Osteopontin (SPP1), Monocarboxylate Transporter 1 (SLC16A1), Membrane-Associated Progesterone Receptor Component 1 (PGRMC1), and Annexin A5 (ANXA5) (Fig. 3c). These proteins defined a profile of unique proteins (*N* = 9) efficiently sorted from donor cells to EVs and enriched at hypoxic conditions, hereafter referred to as “HYP_SIGNATURE_” (the PAN of the replicates of the different peptides is given in Supplementary Fig. 1).

### HYP_signature_ can identify GBM mesenchymal subtype

**Fig. 4:**
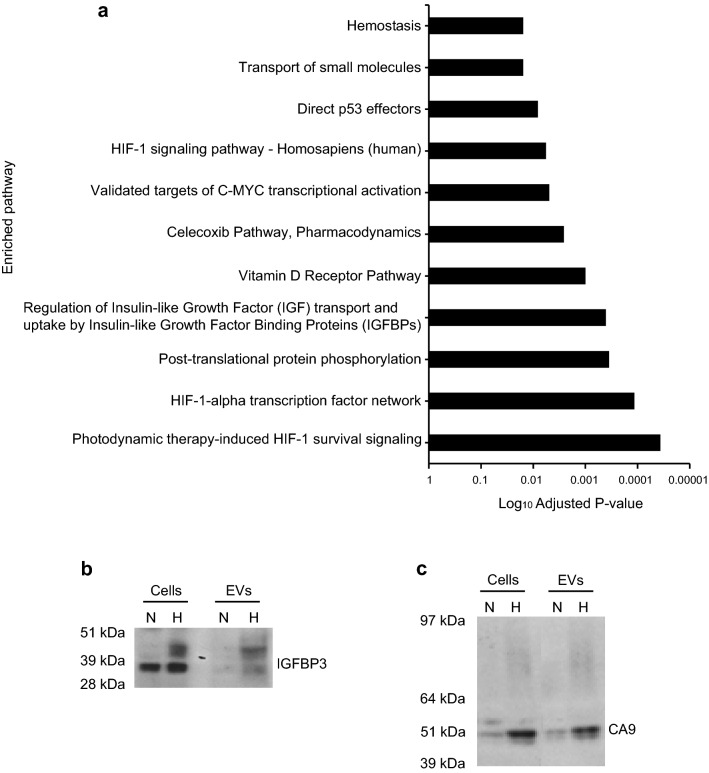
Validation of HYP_SIGNATURE_ top candidate proteins and association with HIF-dependent pathways. **a** Pathway enrichment of HYP_SIGNATURE_ proteins analyzed by ConsensusPathDB-human interaction database. Immunoblotting showing enrichment of IGFBP3 (**b**) and CA9 (**c**) in EV_HYP_ as compared with EV_NORM_ samples

We assayed the pathways enriched by the HYP_SIGNATURE_ proteins using ConsensusPathDB-human interaction database [33]. This identified HYP_SIGNATURE_ to be closely associated with the Hypoxia-Inducible Factor-1α (HIF-1α) transcription factor network (adjusted *P* value = 0.00012) and HIF-1 signalling pathway (adjusted *P* value = 0.0057) with high significance (Fig. 4a). Tissue factor (F3) was previously shown by our group to be enriched in hypoxia-derived EVs [20]. The hypoxic enrichment of other top candidates of the HYP_SIGNATURE_ (Fig. 3c), was supported by immunoblotting, which showed increased levels of IGFBP3 (Fig. 4b) and CA9 (Fig. 4c). Immunoblotting analysis was unable to detect other candidate proteins (NCL, SLC16A1, SPP1, ANXA5) in EVs, either from normoxia or hypoxia (Supplementary Fig. 2b). A potential limitation of these results is the lack of EV housekeeping proteins, and equal protein loading rely on BCA total protein concentration. However, gene array analysis showed increased expression of IGFBP3 (*P* = 0.0012), F3 (*P* = 0.0001), CA9 (*P* = 0.0001), SLC2A1 (*P* = 0.0001), and PGRMC1 (*P* = 0.0017) mRNA in hypoxic as compared with normoxic U87-MG cells (Supplementary Fig. 2a).

**Fig. 5:**
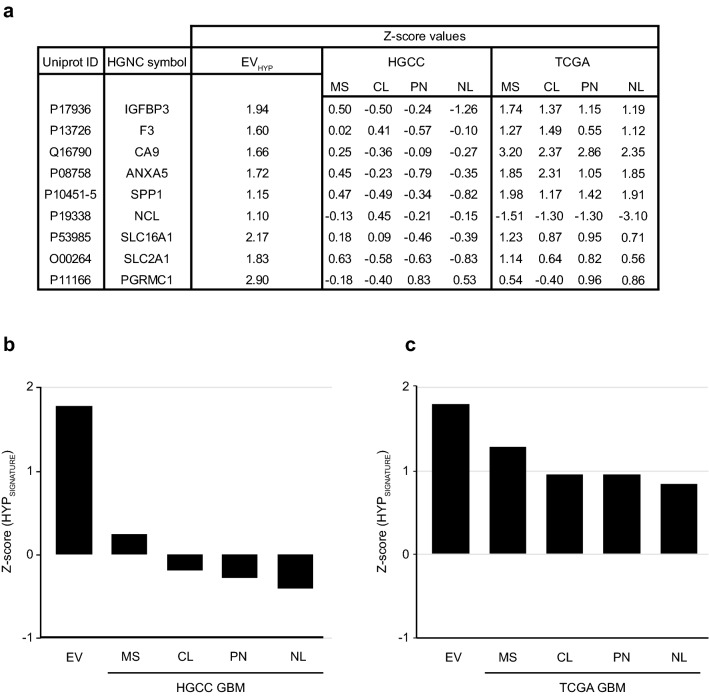
HYP_SIGNATURE_ can identify GBM mesenchymal subtype (**a**) Table showing *Z* score values of HYP_SIGNATURE_ candidates obtained from U87-MG cell EVs, HGCC, and TCGA database. For U87-MG cell EVs, corresponding HYP_SIGNATURE_ candidate protein values from normoxia-derived EVs were used as a reference; for TCGA GBM subtypes, low grade glioma gene expression data of the HYP_SIGNATURE_ candidate genes (*N* = 9) was used as reference value for *Z* score calculations. Bar graph showing relative HYP_SIGNATURE_ cumulative *Z* score comparison between U87-MG cell EVs and HGCC GBM subtypes (**b**), and TCGA GBM subtypes (**c**) respectively. *MS* mesenchymal, *CL* classic, *PN* proneural, and *NL* neural

Several studies have established the association of GBM mesenchymal subtype with hypoxia and an aggressive tumor phenotype [34–36]. To address how the HYP_SIGNATURE_ may associate with the mesenchymal phenotype, we compared the cumulative *Z* score of HYP_SIGNATURE_ with different subtypes of primary GBM cells obtained from Human Glioblastoma Cell Culture (HGCC) *i.e.* classical, proneural, neural and mesenchymal (Fig. 5a). The cumulative HYP_SIGNATURE_*Z* score (1.78) was in close proximity to the HGCC mesenchymal subtype (0.24), evident by their average positive *Z* score as compared with the classical (− 0.18), proneural (− 0.28), and neural (− 0.41) subtypes (Fig. 5b). Next, we compared the HYP_SIGNATURE_ cumulative *Z* score with GBM subtypes obtained from Cancer Genome Atlas Program (TCGA) using the Gliovis portal, which again showed the proximity of HYP_SIGNATURE_*Z* score with the mesenchymal (1.26) as compared with classical (0.94), proneural (0.89), and neural (0.83) GBM subtypes (Fig. 5c).

## Discussion

In this study, we used an optimized combination of nontargeted and targeted quantitative proteomics to comprehensively profile hypoxia-regulated proteins associated with high-grade glioma cell derived EVs. We have identified a protein signature, “HYP_SIGNATURE_”, in EVs secreted by U87-MG cells that is associated with the HIF hypoxic signaling response and exhibited close proximity to the mesenchymal GBM subtype. Importantly, out of the nine proteins encompassing the HYP_SIGNATURE_, seven proteins are known as plasma membrane integrated proteins with an extracellular domain available for specific recognition by antibodies and other targeting agents. Together, our findings thus propose that the hypoxic status of GBM tumors can be defined by the EV HYP_SIGNATURE,_ which may be utilized not only to noninvasively immunephenotype glioma tumors but also as potential therapeutic targets.

The utility of EVs across diverse cellular functions, including recent investigations that support the application of EVs as non-invasive biomarker tools [14, 16, 37, 38], strongly motivates improved efforts to comprehensively profile the proteome of EVs derived from cells grown at disease mimicking conditions. Using discovery proteomics, a previous study [39] identified a total of 844 proteins in EVs isolated from GBM cells. In comparison, we identified approximately 3000 proteins in EVs, out of which 1034 proteins were unique to hypoxic EVs. Importantly, the major aim of the present study was to specifically identify an EV signature that mimics the hypoxic situation, *i.e.* a pathognomonic feature of GBM tumors associated with disease aggressiveness and treatment resistance. Although the studies are limited to one glioma cell-line, it may be argued that the obtained results have general relevance given the substantial overlap between EV protein identities found here and the ExoCarta EV proteome database. Moreover, the hypoxic response is a universal phenomenon of high-grade gliomas as well as other highly malignant tumors. Clearly, future studies will have to further assess the generalizability of the present data, including validation in primary GBM cell models as well as in vivo.

LFQ has now become a widely accepted analytical approach for comparison of the relative abundance of proteins across multiple samples [40–42]. The possibility to analyse untreated proteins or peptides in a large number of samples makes LFQ a preferred protocol over other relative quantification approaches. However, previous studies have shown that sample preparation for the LFQ approach is highly susceptible to variability [43]. Therefore, to reduce this variability, we used 9 replicates of normoxia and 12 replicates of hypoxia samples for LFQ. In addition, the conforming pattern of differential levels of most proteins analyzed in LFQ (Supplementary Table 6) and PRM (Supplementary Table 8), suggest a high degree of sample preparation consistency. In support of EV proteomics data, immunoblotting showed an enrichment of top candidates of the HYP_SIGNATURE_, and gene array analysis showed increased expression of IGFBP3, F3, CA9, SLC2A1 and PGRMC1 mRNA in hypoxic as compared with normoxic U87-MG cells. We were unable to detect other candidate proteins (NCL, SLC16A1, SPP1, ANXA5) in EVs by immunoblotting analysis, either from normoxia or hypoxia, and did not detect a hypoxic enrichment of these proteins in U87-MG cells. A potential explanation to the discrepancy between an induction of these proteins in EVs collected over a cumulative time period of 48 h of hypoxia, and cells analyzed at a fixed time-point, is the well-known temporal dynamics of the hypoxic response.

Several previous studies have associated tumor cell expression of HYP_SIGNATURE_ proteins with increased GBM aggressiveness. For example, F3 expression was demonstrated to be hypoxia-dependent in highly aggressive P7 GBM cells, leading to increased F3 activity [44], and F3-positive EVs were shown to induce angiogenesis [20]. Hypoxia also induced increased SLC16A1 plasma membrane expression in glioma cells, both in in vitro and in vivo models [45]. Additionally, SLC16A1 plasma membrane expression was associated with HIF-1α and CA9 positivity in hypoxic regions. Further, SLC16A1 was found to be upregulated in GBM as compared with normal tissues [46]. NCL was also found to be overexpressed in patient-derived GBM tumors and cells as compared with normal brain [47]. ANXA5 has been found to promote invasion and chemoresistance to the alkylating drug temozolomide in GBM cells [48]. Since hypoxic cells and components in the hypoxic niche have been increasingly implicated in resistance to temozolomide [49], it is conceivable that ANXA5 is associated with the hypoxic component of drug resistance. SPP1 was shown to be induced by hypoxia both in vitro and in vivo [50] and is predominantly observed in the microvasculature of GBM [51]. Several studies have implicated SPP1 with crucial roles in invasion [52] and malignant gliomas [53]. In several glioma cell models, CA9 strongly co-localized with HIF-1α, indicating its induction in hypoxic regions of this tumor type. Clinically, CA9 is minimally expressed in normal brain tissue, whereas its high expression in brain tumors strongly correlated with the level of malignancy [54]. SLC2A1 is another well-established hypoxia-induced protein that has been associated with hypoxic regions of GBM [55]. These studies support a functional role of HYP_SIGNATURE_ protein expression in tumor cells, and future studies that define the tumor promoting role of these proteins when associated with EVs, especially in the context of e.g. pH regulation (CA9), metabolite transport (SLC2A1, SLC16A1), and coagulation activation (F3), will be of high interest.

To conclude, our data strongly support that a specific subset of mostly membrane intercalated EV proteins could define the hypoxic status of high-grade glioma cells. The proteins identified as part of the HYP_SIGNATURE_ warrant further clinical examination using a targeted approach to validate their capacity to differentiate the highly heterogeneous nature of high-grade glioma tumors from e.g. low grade gliomas and other brain lesions that are challenging to define by imaging alone. This proof-of-principle study to noninvasively define the glioma hypoxic status utilizing advanced proteomics is a significant step in this direction.

## Materials and methods

### Cells

U87-MG cells were newly purchased from ATCC. Cells were routinely cultured in DMEM medium, supplemented with 10% foetal bovine serum (FBS), 2 mM L-glutamine, 100 U/mL penicillin and 100 μg/mL streptomycin (growth medium). All cells were grown in humidified 5% CO_2_ incubator at 37 °C. For hypoxia experiments, cells were incubated in humidified Sci-tive NN Hypoxia workstation (Ruskinn Technology) set at 5% CO_2_, 1% O_2_, and 37 °C.

### EV isolation

Normoxic or hypoxic EVs were isolated in parallel from U87-MG cells at a particular passage by standard procedures, using differential ultracentrifugation [20]. Routinely cultured U87-MG cells at sub-confluency were grown in DMEM supplemented with 1% BSA at normoxic or hypoxic conditions for 48 h. Conditioned media were collected after 48 h and centrifuged at 300×*g* twice to eliminate cell debris. Supernatant fractions were then centrifuged at 100,000×*g* for 2 h to pellet EVs, followed by washing twice with PBS at 100,000×*g* for 2 h. EVs were then resuspended in 6 M Urea for downstream proteomics experiments.

### Immunoblotting

U87-MG cells or EV protein lysate were mixed with NuPAGE 4 × LDS Sample Buffer (Life Technologies) and heated for 10 min at 80 °C. Equal amount of proteins was resolved in a NuPage 4–12% Bis Tris gel (Life Technologies) at non-reducing or reducing conditions and then transferred onto a polyvinylidene fluoride (PVDF) membrane (Immobilon-FL), followed by blocking in TBS containing 0.05% Tween 20, 5% nonfat dry milk or 3% BSA for 1 h at RT. To probe for CD9, CD63, CD81, Flotillin-1, IGFBP3, and CA9, the membrane was incubated with the following antibodies in TBST containing 5% nonfat dry milk overnight at 4 °C: anti-CD9 (1:2000; ab92726, Abcam), anti-CD63 (1:100; ab8219, Abcam), anti-CD81 (1:1000; ab109201, Abcam), anti-flotillin-1 (1 µg/mL; ab41927, Abcam), Rabbit anti-IGFBP3 (1:80; PAAJ1, Gro*Pep*), M75 anti-CA9 (1:300; M75, Bioscience Slovakia), Mouse anti-NCL (1:1000, ab13541, Abcam), Rabbit anti-SLC16A1 (1:1000, ab179832, Abcam), Mouse anti-SPP1 (1:500, ab166709, Abcam), and Rabbit anti-ANXA5 (1:500, ab14196, Abcam). After washing, the membrane was incubated with HRP-conjugated anti-mouse IgG (1:10,000) (A9044, Sigma-Aldrich) or anti-rabbit secondary antibody (1:3000) (7074, Cell Signaling Technology). Protein bands were visualized by enhanced chemiluminescence western blotting substrate (Pierce).

Nanoparticle Tracking Analysis, Transmission Electron Microscopy, Trypsin digestion and peptide preparation, Discovery LC–MS/MS, label free quantification, and quantitative LC-PRM-MS/MS were performed as described in Supplementary Materials and Methods.

### Data analysis

The Gene Ontology functional classification of H_significant_ proteins was performed using PANTHER (https://www.pantherdb.org/). Enriched pathways of EV_HYP_ signature proteins were determined using ConsensusPathDB-human interaction database (https://cpdb.molgen.mpg.de/). Wilcoxon test was employed for pathway enrichment analysis with a *P* value cut-off of 0.01.

Gene expression data on different GBM subtypes were obtained from The Cancer Genome Atlas (TCGA) via the GlioVis portal (https://gliovis.bioinfo.cnio.es/), as well as from the Human Glioma Cell Cultures (HGCC) database (https://www.hgcc.se/).

For HYP_SIGNATURE_ comparison in U87-MG cell-derived EVs, the *Z* scores of 9 HYP_SIGNATURE_ candidates were individually calculated for their protein levels with the respective normoxic values as reference as shown by the formula below:

$${\text{Z-score}} = \left( {{\text{EV}}_{{{\text{HYP}}}} {-}{\text{EV}}_{{{\text{NORM}}}} } \right)/\left( {{\text{SD}}\;{\text{EV}}_{{{\text{NORM}}}} } \right)$$

where “EV_HYP_” is the mean protein level measured in hypoxic EVs; “EV_NORM_” is the mean protein level measured in normoxic EVs; and “SD EV_NORM_” is the standard deviation value of the protein level measurements in normoxic EVs. Generation of a cumulative score was done by arithmetic mean of *Z* scores of all 9 HYP_SIGNATURE_ proteins.

For *Z* score calculation on the TCGA dataset, subtype classification of GBM patients was performed with GlioVis portal, and gene expression values for all 9 HYP_SIGNATURE_ candidates were downloaded. Low Grade Glioma (LGG) expression data of the 9 HYP_SIGNATURE_ protein genes was downloaded and used as reference value for *Z* score calculations, as indicated in the formula below:

$${\text{Z-score}} = \left( {{\text{GBM}}\;{\text{subtype}}{-}{\text{TCGA - LGG}}} \right)/\left( {\text{SD TCGA - LGG}} \right)$$

where “GBM subtype” is the mean gene expression value in subtypes such as Classical, Mesenchymal, or Proneural GBM; “TCGA-LGG” is the mean gene expression value for the corresponding gene in LGG patients; and “SD TCGA-LGG” is the standard deviation value of the analyzed gene among the LGG patients. Generation of cumulative score for each GBM subtype was done by arithmetic mean of *Z* scores of all 9 HYP_SIGNATURE_ candidates.

For HGCC data analysis, the gene expression *Z* score for each HYP_SIGNATURE_ candidate in subtypes (Classical, Mesenchymal, Proneural, or Neural) was directly extracted from the HGCC database. Cumulative *Z* score was generated as described for TCGA dataset.

### Statistical analyses

Data are expressed as mean ± STDEV. Statistical analyses were done using unpaired Student *t* test. All values with *P* < 0.05 were considered to be statistically significant.

## Electronic supplementary material

Below is the link to the electronic supplementary material.

Supplementary file1 (DOCX 27 kb)

Supplementary file2 (EPS 1900 kb)

Supplementary file3 (EPS 2000 kb)

Supplementary file4 (XLSX 15 kb)

Supplementary file5 (XLSX 236 kb)

Supplementary file6 (XLSX 230 kb)

Supplementary file7 (XLSX 62 kb)

Supplementary file8 (XLSX 61 kb)

Supplementary file9 (XLSX 329 kb)

Supplementary file10 (XLSX 47 kb)

Supplementary file11 (XLSX 40 kb)
