# Supplementary material for: Global extracellular vesicle proteomic signature defines U87-MG glioma cell hypoxic status with potential implications for non-invasive diagnostics
Source: J Neurooncol. 2019 Aug 14;144(3):477–88. doi: 10.1007/s11060-019-03262-4 (PMC6764937; doi:10.1007/s11060-019-03262-4)
Supplement: Supplementary file 1 — Supplementary file1 (DOCX 27 kb) [file 11060_2019_3262_MOESM1_ESM.docx]

**Supplementary Materials and Methods**

**Global extracellular vesicle proteomic signature defines glioblastoma cell hypoxic status with potential implications for non-invasive diagnostics**

Vineesh Indira Chandran^*^, Charlotte Welinder, Kelin Gonçalves de Oliveira, [Myriam Cerezo-Magaña](http://www.nature.com/ncomms/2016/160420/ncomms11371/full/ncomms11371.html#auth-6), Ann-Sofie Månsson, Maria C. Johansson, Gyorgy Marko-Varga, Mattias Belting

*To whom correspondence should be addressed: Vineesh Indira Chandran, Department of Clinical Sciences, Oncology, Lund, Division of Oncology and Pathology, Lund University, Barngatan 4, SE-221 85, Lund, Sweden; Tel: +46-46-178527; E-mail: vineeshindirachandran[@gmail.com](mailto:mattias.belting@med.lu.se).

ORCID ID: 0000-0003-2160-9379

**Materials and Methods**

***Nanoparticle Tracking Analysis (NTA)***

NTA was applied to determine the size and concentration of particles and to confirm that their size was equivalent to that of EVs [1]. Particles were tracked on an LM10-HS system with a 405 nm laser (Malvern Instruments, Malvern, UK) and visualized with a Luca-DL EMCCD camera (Andor Technology, Belfast, UK). Standard silica beads (0.1-µm) were used to calibrate the analysis settings with a camera level of 10 and detection threshold 2 with blur 9×9. A total of five videos each of 30 s were recorded for the individual samples. Prior to analysis, the samples were diluted in PBS to ensure a particles/frame count within the manufacturer’s recommendations. Particles were tracked, quantitated, and size enumerated using the Nanosight NTA software version 3.0 (Malvern Instruments, Malvern, UK).

***Transmission Electron Microscopy (TEM)***

Approximately 5 µL of isolated EVs were adsorbed onto 400-mesh carbon coated gold grids. The samples were then fixed with 1% glutaraldehyde, stained with 2% uranylacetate and examined in FEI Tecnai BioTWIN TEM operated at an accelerating voltage of 100 KV. Images were recorded with an Olympus SIS Veleta CCD camera.

***Gene Expression Microarray Analysis***

Total RNA was extracted with TRIzol Reagent (Life Technologies) and quantified by using a Nanodrop ND-1000 spectrophotometer (Saven Werner). RNA integrity was verified on an Agilent 2100 Bioanalyzer. Microarray experiments were performed at Swegene Center for Integrative Biology at Lund University Genomics Center at Lund University, Sweden, using one Illumina HumanHT-12 v3 Expression BeadChip. Three independent preparations of normoxic and hypoxic U87 MG cells were analyzed on the 12 arrays on the BeadChip. Data filtration and normalization were performed by using BASE2 [2], and subsequent analyses on transcripts showing a detection *P*-value below 0.01 were performed by using the R statistical programming environment ([www.r-project.org](http://www.r-project.org/)). Hypoxia-mediated gene expression changes, calculated as the ratio of mean hypoxia intensity divided by mean normoxia intensity across the triplicate assays, were determined for cells. Gene lists ranked by the hypoxia/normoxia ratio were used as input for gene set enrichment analysis [3] by using the predefined Curated gene set collection.

***Trypsin digestion and peptide preparation***

Lysed EV samples in 6 M Urea/50 mM ammonium bicarbonate were reduced with 10 mM dithiothreitol for 1 hr at 56^o^C with gentle shaking (300 rpm) and alkylated using 50 mM iodoacetamide for 30 min in the dark. Thereafter, protein samples were digested with sequencing grade trypsin (Promega, Madison) O/N at 37^o^C with gentle shaking (300 rpm). The digestion was stopped by adding 2% trifluoroacetic acid (TFA) (1:10 v/v) and the samples were dried in a Speed Vacuum Subsequently, they were either stored at -80^o^C or resuspended in 0.1% TFA for further analysis.

***Discovery Liquid Chromatography-Mass Spectrometry (LC-MS/MS)***

Approximately 1 µg of hypoxic and normoxia treated samples were analysed by LC-MS/MS with an Easy-nLC II system coupled to a QExactive mass spectrometer (Thermo Scientific, USA). The peptides were concentrated (on-line) by reverse phase chromatography using a 20 mm x 0.75 mm, 3 µm C18 RP precolumn (Acclaim PepMap® 100, nanoViper, Thermo Scientific, USA) and the separated using a 250 mm x 0.075 mm, 2 µm (Acclaim PepMap® 100 RSLC, nanoViper, Thermo Scientific, USA) with a flow rate of 300 nL/min. The peptides were eluted using a nonlinear gradient. The gradient started with 5% B and 40% B at 90 min, followed by 90% B at 95 min which was maintained for 10 min (solvent A: 0.1% formic acid and solvent B: 0.1% formic acid in ACN). Peptides were detected using top15 data-dependent approach. Full MS scans were acquired in the Orbitrap mass analyzer over m/z 400–1600 range with resolution 70,000. The target value was 1.00E+06. The fifteen most intense peaks with charge state ≥ 2 were fragmented in the HCD collision cell with normalized collision energy of 30%, and tandem mass spectra were acquired in the Orbitrap mass analyzer with resolution 17,500 at m/z 200. The target value was 1.00E+06. The ion selection threshold was 3.30E+04 counts, and the maximum allowed ion accumulation times were 100 ms for full MS scans and 60 ms for tandem mass spectra. For all the experiments, dynamic exclusion was set to 30 s.

Raw files were analyzed with Proteome Discoverer v 1.4 (Thermo Scientific, USA). Peptides were identified using SEQUEST HT against UniProtKB human database [4] integrated into Proteome Discoverer. The search was performed with the following parameters applied: carbamidomethylation as static modification, oxidation of methionine as dynamic modification, 10 ppm precursor tolerance and 0.02 Da fragment tolerance. Up to one missed cleavage for tryptic peptides was allowed. Filters: ‘high confidence’ and ‘at least two peptides per protein’ were applied (FDR ≤ 0.01).

***Label free quantification (LFQ)***

For LFQ of proteins detected by discovery LC-MS/MS, raw PD files were subjected to a typical processing and consensus workflow in Proteome Discoverer (PD) version 2.2 delivered by Thermo Fisher Scientific. In this script for LFQ, firstly the LC/MS peaks in the raw PD data files were detected using Minora Feature Detector and mapped to identified peptide spectrum matches (PSMs). Thereafter, the algorithm created features from unique peptide-specific peaks that were within a small retention-time range using Retention Time Aligner and Feature Mapper. The identification and quantification were performed with the following settings applied; Peptides with a minimum length of 6 amino acids were identified with high confidence, target FDR set at 0.01. For chromatographic alignment, maximum retention time shift allowed was 10 min and 10 ppm mass tolerance. For feature linking and mapping, minimum hypoxia vs. normoxia threshold was set at 5. Quantification abundances of the identified unique peptide precursor ions were normalized to the same total peptide amount per channel and scaled, so that the average abundance per protein and peptides is 100.

***Quantitative Liquid Chromatography-Parallel Reaction Monitoring-Mass Spectrometry (LC-PRM-MS/MS)***

MS1 chromatogram-based quantification was performed in Skyline version 3.1, <http://proteome.gs.washington.edu/software/skyline> [5]. A spectral library was established by importing the MSF files generated within Proteome Discoverer into Skyline. Then, raw data files were directly imported into Skyline, and MS1 precursor ions extracted (match tolerance of 0.5 m/z) for all peptides presented in the MS/MS spectral libraries. An isolation list with precursors for the 402 peptides (135 protein groups) was generated for an unscheduled LC-MS/MS run. The raw files were directly imported into Skyline and an isolation list with the precursors, selected transitions and with retention time window of 4 min was generated for a new scheduled LC-MS/MS run. The peptides, its precursor mass and the retention time of the protein groups used for the scheduled run are given in Table S7. The three highest intensity fragment ions (y-ions) were selected for PRM transitions for each peptide. The peak normalized areas (PAN) of individual PRM transitions were then summed to obtain the total PAN of each peptide and compared between normoxic and hypoxic treatment conditions. PRM parameters were optimized and selected from prior experiments.

**References**

1. Mork M, Handberg A, Pedersen S, Jorgensen MM, Baek R, Nielsen MK, and Kristensen SR (2017) Prospects and limitations of antibody-mediated clearing of lipoproteins from blood plasma prior to nanoparticle tracking analysis of extracellular vesicles. J Extracell Vesicles. 6: 1308779.

2. Vallon-Christersson J, Nordborg N, Svensson M, and Häkkinen J (2009) BASE - 2nd generation software for microarray data management and analysis. BMC Bioinformatics. 10: 330.

3. Subramanian A, Tamayo P, Mootha VK, Mukherjee S, Ebert BL, Gillette MA, Paulovich A, Pomeroy SL, Golub TR, Lander ES, and Mesirov JP (2005) Gene set enrichment analysis: A knowledge-based approach for interpreting genome-wide expression profiles. Proceedings of the National Academy of Sciences. 102: 15545-15550.

4. (UniProt Consortium (2015)) UniProt: a hub for protein information. Nucleic Acids Res. 43: D204-12.

5. Prakash A, Tomazela DM, Frewen B, Maclean B, Merrihew G, Peterman S, and Maccoss MJ (2009) Expediting the development of targeted SRM assays: using data from shotgun proteomics to automate method development. J Proteome Res. 8: 2733-9.
